# Chamber Protection of Zinc with Ethylhexanoic Acid

**DOI:** 10.3390/ma16103679

**Published:** 2023-05-11

**Authors:** Olga A. Goncharova, Andrey Yu. Luchkin, Nickolay N. Andreev, Oleg Yu. Grafov, Olga S. Makarova, Ilya A. Kuznetsov, Sergey S. Vesely

**Affiliations:** Frumkin Institute of Physical Chemistry and Electrochemistry, Russian Academy of Sciences, 119071 Moscow, Russia; skay54@yandex.ru (A.Y.L.); n.andreev@mail.ru (N.N.A.); grafov.oleg88@gmail.com (O.Y.G.); olga.byvsheva.99@mail.ru (O.S.M.); anarenen@gmail.com (I.A.K.); sergei57@mail.ru (S.S.V.)

**Keywords:** zinc, atmospheric corrosion, chamber corrosion protection, chamber inhibitors, ethylhexanoic acid

## Abstract

Chamber protection is a promising and quickly developing method of vapor-phase protection of metals against atmospheric corrosion by inhibitors. It was shown that chamber treatment with 2-ethylhexanoic acid (EHA) efficiently inhibits the initiation of zinc corrosion. The optimum conditions (temperature and duration) of zinc treatment with vapors of this compound were determined. If these conditions are met, adsorption films of EHA with thicknesses up to 100 nm are formed on the metal surface. It was found that their protective properties increase during the first day as zinc is exposed to air after chamber treatment. The anticorrosive action of adsorption films is due both to the surface being shielded from the corrosive environment and to the inhibition of corrosion processes on the active surface of the metal. Corrosion inhibition was caused by the ability of EHA to convert zinc to the passive state and inhibit its local anionic depassivation.

## 1. Introduction

The global losses from corrosion are measured in trillions of USD annually [1]. Atmospheric corrosion accounts for 60% of these losses [2]. Vapor-phase protection of metals via inhibitors is a reliable and cost-effective approach to combat it [3,4,5,6]. Since the middle of the 20th century, volatile corrosion inhibitors (VCIs) have been widely used in the industry in developed countries [7,8,9]. A distinctive feature of VCIs is that they have a high vapor pressure. As a VCI evaporates at ambient temperature, it saturates the space, reaches the metal as vapor, adsorbs on its surface, and provides reliable protection of the product.

A newer method of vapor-phase protection of metals, suggested and developed by us, involves so-called chamber inhibitors (CINs) [10,11,12]. Unlike VCIs, their volatility under ordinary conditions is low. The chamber protection (CP) of metals is as simple as a short exposure of a metal to vapors of CINs in a closed space (chamber) at an elevated temperature. Provided that the inhibitor and treatment temperature are chosen properly, nanosized adsorption films are formed on the metal surface. They are capable of protecting the metal under outdoor conditions for a long time after it has been withdrawn from the chamber. In this case, CP has significant advantages over the traditional vapor phase protection by VCIs. Chamber protection has the following features:

- It does not require sealing the product to be protected and the inhibitor together for the entire preservation period;
- It consumes a very small amount of inhibitors. In fact, a CIN is only consumed for the formation of adsorption layers on the metal item and on the chamber walls;
- It considerably expands the scope of inhibitors that can be used for vapor-phase protection by including low-volatile compounds that cannot be utilized as VCIs;
- It is environmentally friendly. There are no waste products typical for VCIs that harm the environment, such as used-up emitters, packaging materials, etc.;
- It is safe for humans. Metals are treated in an airtight chamber, so contact of the CINs with the personnel engaged in the preservation activities is prevented.

Previously we considered the capabilities of the method and the regularities of the CP of items made of steel, copper, brass, magnesium, and some other materials [10,11,12]. This work deals with the CP of zinc by individual compounds, primarily 2-ethylhexanoic acid (EHA).

## 2. Materials and Methods

### 2.1. Materials

The protective properties of CINs, and the thickness and structure of the surface films were studied on Ts0 zinc manufactured in Russia. The metal had the following composition (Table 1) [13]:

Corrosion screening was performed by analyzing the protective properties of octadecylamine, diphenylguanidine, polyethylene polyamine, hexamethylenetetramine, Captax, Altax, benzotriazole, lauric, tridecane, stearic, oleic, and linolenic acids, and EHA. The following additional reagents were used: acetone, sodium tetraborate, boric acid, calcium chloride, and sodium chloride. All the reagents were commercial products of “pure” or “chemically pure” grades. Solutions were prepared with distilled water.

### 2.2. Preparation of Samples and Electrodes

Corrosion and electrochemical experiments were performed using flat rectangular zinc samples, 30 × 40 × 3 mm in size with holes for mounting in cells and chambers. They were cleaned with sandpaper of various grit sizes (from 240 to 1500), degreased with acetone, and stored in a desiccator with calcined CaCl_2_ for at least 3 days. In ellipsometric and XPS studies, samples 8 × 8 × 2 mm in size were used. In addition to sandpaper treatment, they were polished on felt with a diamond paste containing particle sizes from 3 to 0.25 μm, and then degreased. After polishing, the diamond paste residues were removed from the surface using repeated ultrasonic washing with acetone.

Chamber treatment (CT) and thermal treatment (TT) of zinc were performed in 0.6 L glass cells with the inhibitor (0.1 g) or without it. Samples were hanged in the cells on nylon threads. Next, the cells were hermetically sealed and placed in a heated drying oven. The temperature of the oven and the exposure time of the cells are specified in the text below.

### 2.3. Corrosion Methods

In the corrosion experiments, the time until the first corrosion damage on the metal appeared was recorded.

#### 2.3.1. Tests with Recurrent Moisture Condensation

Metal samples were hung from the caps of sealed glass cells on nylon threads so that they did not come into contact with each other or with the cell walls. The volume of each cell was 0.6 L. A 0.1 L portion of water with a temperature of 50 °C was poured into each cell, which caused intense moisture condensation on the samples. Once a day, water was replaced with a new portion of hot water. The samples were visually inspected through the transparent cell walls every hour for two days from the beginning of the test. After this period, they were inspected every 6 h.

#### 2.3.2. Salt Spray Tests

The tests in neutral salt spray were conducted in a WEISS SC 450 chamber at room temperature. Each one-hour test cycle included a 15 min period of spraying with 3% NaCl solution and a 45 min exposure of the samples to the salt spray that formed. The samples were inspected 55 min after the start of each cycle.

#### 2.3.3. Field Tests

Field tests of CIN efficiency were performed at the Moscow corrosion station. Zinc samples prepared using one of the methods described above were placed vertically on test stands in a louvered booth. The samples were inspected once a week.

### 2.4. Electrochemical Methods

#### 2.4.1. Voltammetry

Anodic polarization curves were recorded using an IPC-Pro MF potentiostat (Russia) in potentiodynamic mode at a sweep rate of 0.2 mV/s. The experiments were performed in a clamp-on electrochemical cell. The surface area of the working electrode was 1 cm^2^. The auxiliary electrode was made of platinum. The reference electrode was a saturated silver chloride electrode. Potential (*E*) values were converted to the normal hydrogen scale. Experiments were performed at room temperature with natural electrolyte aeration. A borate buffer solution (BBS) with pH 7.36 containing 1 mM NaCl served as the electrolyte.

To perform the experiments, the cell was mounted on a flat electrode sample, filled with electrolyte, kept for 15 min, and polarized anodically from the stationary potential (*E*_st_). Analysis of polarization curves focused on the effect of inhibitors on the current density (i) of active dissolution and the breakdown potential of the passive film (*E*_br_). *E*_br_ was determined from a sharp rise in current with mandatory subsequent visual identification of pits on the samples. The state of the metal surface was monitored using an MBS-2 microscope.

#### 2.4.2. Electrochemical Impedance Spectroscopy

The efficiency of various zinc treatment modes and the mechanisms of its protection with CINs were analyzed using electrochemical impedance spectroscopy. For this purpose, a potentiostat of the same brand as in voltammetric experiments, additionally equipped with an FRA-2 frequency response analyzer module (Russia), was used. The design of the cell and the model electrolyte were the same as those described above. Impedance measurements were carried out in potentiostatic mode at *E*_st_ using superposition of a harmonic signal with an amplitude of 10 mV in a frequency range of 0.1–10^5^ Hz.

### 2.5. Ellipsometry

The thicknesses of the films formed on metals under specific conditions were measured with a manual Gartner ellipsometer with light beam modulation and advanced recording of light emission. An LSM-S-111-10-NNP25 solid-state diode-pumped laser with a wavelength of 540 nm was used as the source. In the experiments, the variation in the ellipsometric angles Δ and Ψ was recorded.

δΔ = Δ − Δ_0_, δΨ = Ψ − Ψ_0_, (1)

where the angles Δ_0_ and Ψ_0_ correspond to the values of the initial surface state, while Δ and Ψ are the current values obtained after the surface state changed due to chemisorption of CIN molecules and/or oxide growth. The thicknesses of inhibitor and oxide films were determined using the Ellipsometry Calculation Spreadsheet program [14] in the three-layer model approximation.

In the experiments for determining CIN film thicknesses, zinc samples prepared as described above were mounted vertically on the measuring table of the ellipsometer.

### 2.6. X-ray Photoelectron Spectroscopy

The studies were performed using an OMICRON ESCA+ spectrometer (Germany) with an aluminum anode equipped with an XM1000 monochromatic X-ray source (AlKα, 1486.6 eV, 252 W power). A CN-10 charge neutralizer with an emission current of 4 μA and a beam energy of 1 eV were used to eliminate the local charge on the surface being analyzed. An Argus analyzing detector was used. The analyzer pass energy was 20 eV. The spectrometer was calibrated using the Au4f 7/2 line at 84.1 eV. The pressure in the analyzer chamber did not exceed 10^−9^ mbar. All the spectra were measured at least in triplicate. The background was subtracted using the Shirley method [15].

## 3. Results

### 3.1. Corrosion Screening

The ability of individual chemical compounds of different nature to provide chamber protection of zinc was estimated under periodic moisture condensation conditions. CT was performed at a temperature (*t*_CT_) of 120 °C; its duration (*τ*_CT_) was 1 h. After CT, the samples were exposed to ambient conditions for 24 h (τ_exp_) before placement in the corrosive medium. These conditions have previously been suggested for the corrosion screening of CINs [16]. Note that they are not optimal for many of the compounds studied; however, they allow to perform an initial selection of promising inhibitors.

During experiments of this type, the first indications of zinc corrosion (dots) appeared 1 h after hot water was poured into the cells on samples that had not been subjected to chamber treatment or TT. TT without a CIN did not affect the corrosion resistance of zinc. The incubation time of corrosion (*τ*_cor_) was still no more than 1 h.

All the compounds studied inhibited the initiation of corrosion to some extent (Table 2). However, the protective aftereffect (PAE) of CINs varied in a wide range. In the case of amines with various structures, *τ*_cor_ ranged from 2 h for diphenylguanidine to 12 h in the case of polyethylene polyamine. The *τ*_cor_ in the case of heterocyclic compounds such as Captax, Altax, and benzotriazole was 4, 2, and 8 h, respectively.

Carboxylic acids with a linear hydrocarbon chain were more efficient. Their PAE varied from 24 h for lauric and stearic acids to 48 h for tridecanoic acid. At the same time, the length of the hydrocarbon chain did not strongly affect the protective properties. For example, lauric (C_11_H_23_COOH) and stearic (C_17_H_35_COOH) acids had equal *τ*_cor_ values. At the same time, tridecanoic acid (C_12_H_25_COOH) that had almost the same length of the hydrocarbon radical as lauric acid, protected zinc twice as long.

The presence and number of double bonds had no noticeable effect on the PAE either. This follows from a comparison of the following *τ*_cor_ values: 24 h for stearic acid (no double bonds), 12 h for oleic acid (one double bond), and 12 h for linolenic acid (three double bonds). EHA was considerably superior to the other carboxylic acids in terms of PAE. It completely protected zinc for 1680 h. We can assume that this results from the branched hydrocarbon chain. This assumption is indirectly confirmed by the high anti-corrosion properties of neodecanoic acid. We will not consider this compound, which is a mixture of branched carboxylic acids, in detail in this work that deals with individual compounds, but it may be noted that its protective aftereffect also exceeded 1600 h.

Thus, corrosion screening made it possible to single out EHA as a promising CIN against zinc corrosion from other compounds. The following sections deal with the analysis of the protective properties of EHA.

### 3.2. Optimization of the Conditions of Zinc CT with EHA in Terms of Temperature and Duration

As we noted previously, each “metal–CIN” system is characterized by its optimal CT mode [10,11,12]. In this context, the effect of *t*_CT_ and *τ*_CT_ on the PAE of EHA on zinc was studied. Taking into account that the experiments with recurrent moisture condensation take a very long time, this part of the study was carried out under more drastic salt spray conditions.

For zinc in the initial state, corrosion damage already appears on samples after the first test cycle of salt spray. This is comparable with the results obtained in blank tests with recurrent moisture condensation. However, the PAE of the CIN under recurrent moisture condensation conditions is considerably longer than the aftereffect in salt spray. Therefore, corrosion tests of this type provided significant time saving.

In the first series of experiments in salt fog we analyzed the effect of *t*_CT_ on the τ_pr_ of EHA. The data in Table 3 show that TT in the absence of a CIN fails to increase the corrosion resistance of zinc. Regardless of the TT temperature, the first indications of corrosion on zinc (white spots) appeared during the first test cycle. Following addition of EHA to the chamber τ_prot_ increased, with an increase in the *t*_CT_ of zinc from 20 to 100 °C by almost two orders of magnitude (up to 72 h). According to [10,11,12], this is due to the increase in the EHA vapor pressure in the chamber, which favors the adsorption of the inhibitor. At higher temperatures (up to 120 °C), heating of the chamber decreased the protection efficiency. The descending branch of the temperature dependence of EHA’s PAE may be associated with a decrease in adsorption as the temperature of the adsorbent (zinc) increases.

Thus, the optimal *t*_CT_ value is about 100 °C. This temperature was used to create adsorption films in subsequent studies on the protective properties of EHA on zinc, including the second series of salt fog tests where the optimal CT duration was determined. The dependence of τ_pr_ on *τ*_CT_ presented in Table 4 indicates that the PAE of EHA increases to 72 h during the first hour of chamber treatment. Any subsequent exposure of zinc in a chamber with EHA did not amplify protection and was inexpedient.

Thus, 1 h is the optimal *τ*_CT_ value. In our subsequent studies of the protective properties and mechanisms of zinc CP with EHA, the adsorption films were formed using CP for this period of time. The high efficiency of zinc CP with EHA is confirmed by the results of outdoor tests presented in Table 5. During exposure of zinc in the initial state and after TT without a CIN at the Moscow Corrosion Station, metal tarnishing was already noticeable in the first inspections. CT with EHA provided full metal protection for more than 42 months. At the time this article was written, there was no corrosion damage on the zinc samples treated in this way.

### 3.3. Mechanisms of Zinc CP with EHA

**Figure 1:**
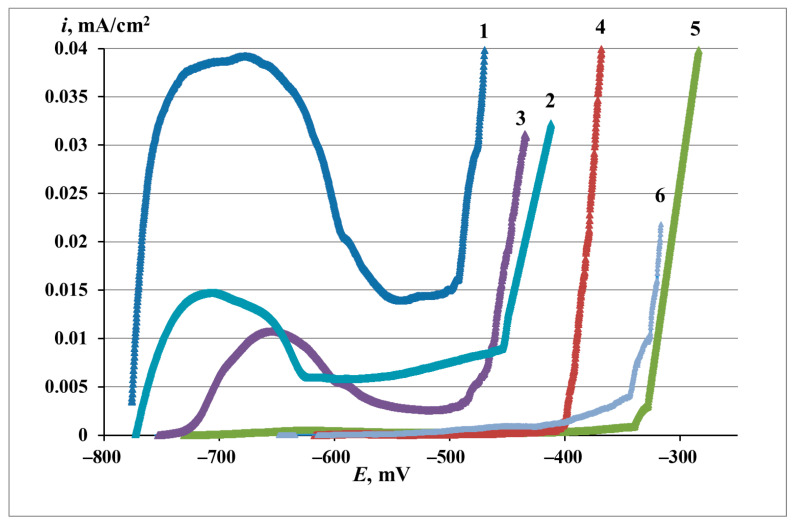
Anodic polarization curves of zinc (BBS, pH 7.36, 1 mM NaCl) **in the initial state (1)**, **after TT without a CIN**, ***τ*_exp_ = 24 h (2)**, and after CT in EHA vapors with various *τ*_exp_: (3)—1 h; (4)—5 h; **(5)—24 h**; (6)—72 h. *t*_CT_ = 100 °C; *τ*_CT_ = 1 h.

The above data of corrosion experiments indicate the high protective properties of EHA in CP and allow to determine the optimum conditions of zinc treatment with this. However, they do not give even approximate information about the mechanisms of action of this CIN. In view of this, the effect of EHA on the anodic dissolution of zinc was studied. The anodic polarization curves of zinc that underwent different modes of treatment are shown in Figure 1. Their characteristic parameters are given in Table 6. The experiments analyzed in this part of this study are marked with bold letters in the caption of the Figure and in Table 5.

The anodic polarization curve of zinc in initial state (curve 1) comprises an active dissolution region and a passive region followed by local breakdown of passivity, with the following characteristic values: *E*_st_ = −0.770 V, *i*_pas_ = 49 µA, *E*_br_ = 0.520 V (where *i*_pas_ is the passivation current density). The current density in the passive area is rather high and does not fall below 15 µA/cm^2^. After the experiment, one or more dark spots, i.e., pits, could be observed on the zinc surface.

The anodic polarization curve of zinc after TT without a CIN (curve 2) is close to curve 1 in appearance and characteristic values. TT inhibited the anodic dissolution. This manifested in a slight ennoblement of *E*_br_ and a decrease in the passivation current.

CT with EHA under the optimal conditions (curve 5) led to zinc passivation. *E*_st_ was ennobled by about 0.045 V. The *i* values in the passive region decreased to about 1–2 μA/cm^2^. The *E*_br_ shifted by about 0.165 V to the anodic region. Inspection of the surface confirmed the local mode of metal depassivation. Thus, comparison of polarization curves 1, 2, and 5 indicates that the protective effect of EHA is associated with the passivation of zinc and stabilization of its passive state.

**Figure 2:**
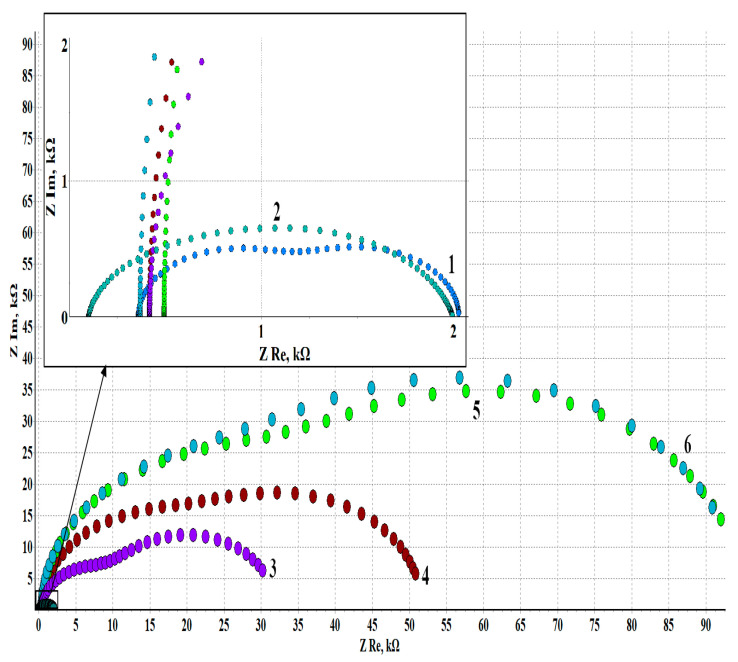
Nyquist plots obtained in BBS (pH 7.36, 1 mM NaCl) **in the initial state (1), after TT without a CIN, *τ*_exp_ = 24 h (2)**, and in EHA vapors with various *τ*_exp_: (3)—1 h; (4)—5 h; **(5)—24 h**; (6)—72 h. *t*_CT_ = 100 °C; *τ*_CT_ = 1 h.

Electrochemical impedance spectroscopy provides additional information about the protection mechanism. The Nyquist plots of zinc electrodes treated in various modes are shown in Figure 2, and the values of the elements of the equivalent circuit describing the electrode processes are listed in Table 7. The experiments analyzed in this part of the work are shown in bold letters in the caption of Figure 1 and in Table 7.

It is clearly seen that all the hodographs are arcs (or fragments) of semicircles. At the same time, the radius of the arcs increases manyfold for zinc electrodes treated with EHA vapors. This means that CIN increases the resistive properties of the metal surface and hinders the corrosion process.

**Figure 3:**
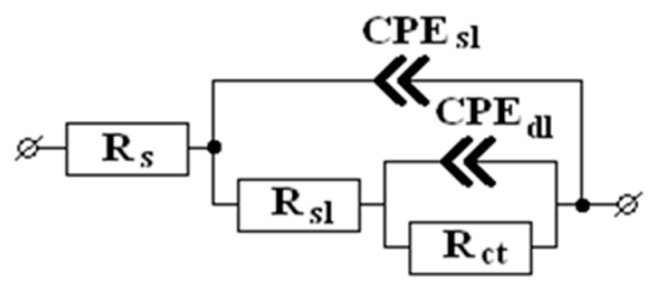
Equivalent circuit used in the study.

The electrochemical impedance spectra obtained in this way were adequately approximated using a modified Mansfeld equivalent circuit [17,18,19] where the capacitive elements were replaced by constant phase elements (*CPE*): the experimental data matched the calculated data to at least 98% (Figure 3). This type of circuit is widely used to simulate the corrosion and electrochemical behavior of metals with porous coatings and/or films (Figure 1) [20,21].

In this scheme, *R*_s_ is the resistance of the electrolyte between the test sample and the reference electrode’s capillary. It depends on the solution conductivity and the distance between the sample surface and the capillary and does not affect the electrode processes. *R*_sl_ is the sum of resistances of the oxide–hydroxide and adsorption surface layers, and *R*_ct_ is the charge transfer resistance of the Faraday reaction that determines the kinetics of the corrosion process. *CPE*_sl_ is the constant phase element that characterizes the capacitance of the metal–electrolyte interface and mainly depends on the structure of the surface layers. *CPE*_dl_ is the constant phase element that reflects the capacitance of the electrical double layer in the Faraday reaction.

The impedance of the constant phase element is described by equation [22]:

*Z*_CPE_ = *Q*^−1^(*jω*)^−*n*^, (2)

where *Q* is the element modulus, *n* is the phase factor, *j* is the imaginary unit, and ω is the cyclic frequency. At *n* = 1, the modulus of the CPE element has the properties of a perfect capacitor. As the value of *n* decreases, the effects of heterogeneity of the capacitor plates and diffusion processes on the behavior of the constant phase element gradually become stronger, and at *n* = 0.5 this element corresponds to a Warburg element. Thus, the values of *Q* and phase factor *n* of the CPE elements make it possible to gain additional information about the character of electrode processes and the surface state. The values of the equivalent circuit elements were calculated using the Dummy Circuits Solver Version 2.1 program.

The degree of protection of zinc electrodes after CT was calculated as follows:

*Z* = (*R*_CT_ − *R*_TT_)/*R*_TT_ × 100%, (3)

where *R*_CT_ is the total of the active resistances *R*_CT_ and *R*_sl_ in the equivalent circuit for a zinc sample that underwent CT. Similarly, *R*_TT_ is the sum of *R*_CT_ and *R*_sl_ for the tests where the metal underwent TT without a CIN.

It follows from the data in Table 7 corresponding to hodographs 1, 2, and 5 that the *CPE*_sl_ element is a perfect capacitor because its exponential phase factor is equal to 1 or close to it. Hence, the *CPE*_sl_ modulus can be presented in units of capacitance, F/cm^2^, for convenience. It can be seen that the highest capacitance (5.19 μF/cm^2^) is observed for zinc in the initial state. After TT, the capacitance becomes two times smaller (2.37 μF/cm^2^). CT decreases the sample capacitance even more, to 1.90 μF/cm^2^. The decrease in the capacitance of the *CPE*_sl_ element upon TT is most likely caused by an increase in the thickness of the surface oxide that plays the role of a dielectric spacer in the capacitor. This correlates well with the increase in the value of *R*_sl_ that simulates the resistance of the surface oxide in the equivalent circuit. TT results in some growth of the oxide phase on the surface, as *R*_sl_ increases by about 20%. Treatment with EHA vapors leads to a sharp, approximately 50-fold increase in the resistance of the surface films. This is due not so much to surface oxidation as to EHA adsorption on the surface.

The *CPE*_dl_ and *R*_ct_ elements correspond to the electrochemical process on the zinc surface under an oxide layer. It can be seen that the charge transfer resistance *R*_ct_ changes little after the TT of zinc but increases manyfold (more than 50-fold) after CT. In other words, not only mechanical surface blocking but also inhibition of the Faraday process occurs if a CIN is used. The parameters of the electrical double layer (judging by the *CPE*_dl_ modulus and the phase factor *n*_dl_) do not change so significantly and remain in the range of 67–190 μF/cm^2^.

It is noteworthy that as zinc electrodes are kept in the air after CT, the values of *R*_sl_ and *R*_dl_ increase considerably with time. The value of *R*_sl_ increases in 24 h while *CPE*_sl_ decreases approximately 3.5-fold compared to the samples exposed to air for 1 h. The surface film undergoes some changes leading to a growth in corrosion resistance, but it remains homogeneous, as the *n*_sl_ value confirms.

Moreover, *R*_CT_ increases approximately 2.5-fold, while *CPE*_ct_ decreases by an order of magnitude. Thus, we can state that the fraction of the electrochemically active surface decreases due to some structurization of the film on the zinc surface after the CT. The inhibition coefficient *Z* = 98.24% confirms the high efficiency of the CIN.

The results of simulating experimental data using the equivalent circuit make it possible to numerically estimate the contribution of various mechanisms that provide the inhibitory effect of EHA and determine the partial corrosion inhibition coefficients.

Two main mechanisms of action of corrosion inhibitors of the adsorption type are known: blocking and activation [23,24]. In the first case, as the inhibitor is adsorbed, it blocks a fraction of the metal surface, thus reducing the corrosion rate, but does not affect the kinetics of electrode processes on the remaining surface that is not blocked. In contrast, the activation mechanism implies corrosion inhibition by altering the activation energy of corrosion processes, and, hence, their kinetics. Both mechanisms usually operate simultaneously, but their contributions to the inhibition effect may differ.

The *R*_sl_ value in the equivalent circuit reflects the effect of the surface layer and can therefore serve as a criterion for estimating the blocking effect of the inhibitor. The coefficient of corrosion inhibition due to surface blocking (*γ*_sl_) equals the ratio of resistance *R*_sl_ of the sample after CT to *R*_sl_ after TT without a CIN:

*γ*_sl_*= R*_sl_^CT^*/R*_sl_^TT^. (4)

Using a similar approach, the effect of the CIN on the Faraday corrosion process can be judged from the *R*_ct_ value. The coefficient of electrochemical reaction inhibition due to a CIN (γ_ct_) can be determined as the ratio of the charge transfer resistances *R*_ct_ for inhibited and uninhibited samples:

*Γ*_ct_*= R*_ct_^CT^*/R*_ct_^TT^. (5)

The degrees of protection by a CIN via the blocking and activation mechanisms in different modes of zinc CT are shown in Table 8. It follows from the data in Table 8, corresponding to τ_exp_ = 24 h, that *γ*_sl_ and *γ*_ct_ are significantly greater than one and comparable in magnitude. This indicates that EHA acts via a mixed blocking–activation mechanism.

The protective effect of EHA results from the formation of nanoscale adsorption films on the surface. This is confirmed by the ellipsometry data presented in Table 9. As above, the experiments analyzed in this part of the work are shown in bold in Table 9.

TT in the absence of EHA results in the growth of the oxide film on zinc surface. It becomes thicker by ca. 5.5 nm, regardless of the treatment time. Note that this result confirms the assumption about the reasons for the decrease in the *CPE*_sl_ capacitance of a zinc electrode upon TT made in the discussion of electrochemical impedance spectra. CT of zinc under optimal conditions inhibits zinc oxidation. In this case, the oxide layer thickness increases by only 1 nm. However, an adsorption layer of the CIN, ca. 85 nm thick, is formed on the surface.

Thus, the chamber protection of zinc by EHA is due to the formation of nanosized adsorption surface films that passivate the metal and stabilize the passive state. The corrosion inhibition of zinc can be characterized by a mixed blocking–activation mechanism.

### 3.4. Structuring of the Surface Films Formed upon Zinc CT with EHA

An interesting experimental fact noted in the electrochemical studies of the “zinc–EHA” system is that the protective properties of surface films grow during the first day of exposing the electrodes to air at room temperature after the CT. The self-organization process of protective layers is illustrated by the data in Figure 2 and Figure 3 and in Table 6 and Table 7.

Analysis of polarization curves shows that as τ_exp_ grows, the *i*_pas_ values decrease, up to complete passivation of the metal, while *E*_br_ becomes more positive. At τ_exp_ = 1 h, the *i*_pas_ value was 5 μA/cm^2^, while within the next 4 h of exposure to air (τ_exp_ = 5 h) the electrode passivated. The *E*_br_ value corresponding to τ_exp_ = 1 h was −0.465 V. After 5 and 24 h of electrode exposure to air, the passive film breakdown was recorded at −0.405 and −0.340 V, respectively.

Exposure of electrodes to the air after CT for more than 24 h was not accompanied by changes in the basic parameters of polarization curves that characterize the CP efficiency. The increase in the protection efficiency also follows from the analysis of electrochemical impedance spectra. The *Z* value corresponding to 1 h of electrode exposure outside the chamber was 94.7%. The degree of protection increased to 96.7 with an increase in τ_exp_ to 5 h and to 98.2% after 24 h. One day after the CT, the protective capacity of the adsorption films stabilized.

The mechanism of EHA protective action did not change with exposure. The data in Table 8 indicate that it can be characterized as a mixed blocking–activating mechanism over the entire τ_exp_ range studied.

It is important that the structuring of surface films described above was not accompanied by film thickening (Table 9). Moreover, the thickness of the adsorption layers decreased from 105 to 85 nm during the first day.

The combination of these data suggests the following general scheme of the process. During the CT, EHA is adsorbed on the zinc surface. Even during this treatment, some fraction of the inhibitor reacts with the metal and with the surface oxide, creating compounds with some protective properties. After the electrode is removed from the chamber and kept in open air, the unreacted EHA evaporates into the atmosphere, which can cause thinning of the surface film. At the same time, reactions of formation of surface compounds that protect zinc continue to occur, so its corrosion resistance continues to increase.

**Figure 4:**
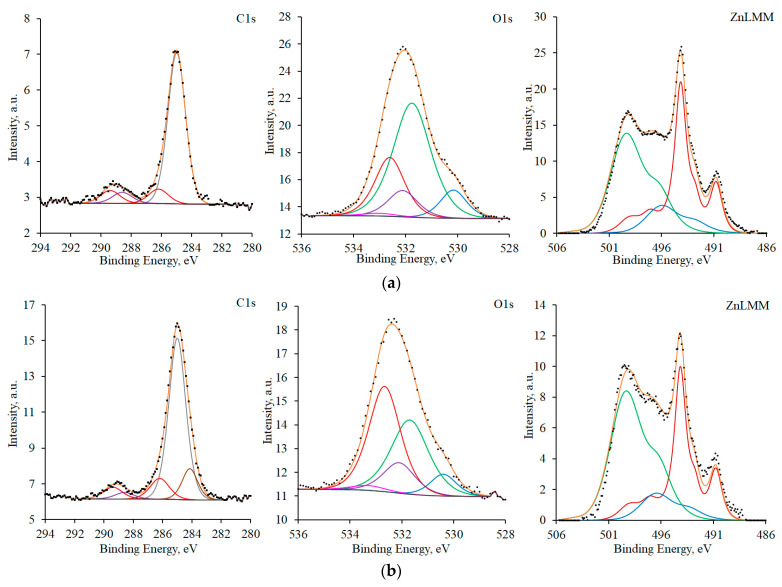
C1s and O1s XPS spectra, and ZnL_3_M_45_M_45_ Auger spectra for samples in the initial state (**a**) and after TT without a CIN (**b**). Spectral components are given in different colors for better visibility.

XPS data are useful for clarifying this rather general picture. Figure 4 presents the C1s and O1s XPS spectra, along with the ZnL_3_M_45_M_45_ Auger spectra for samples before TT and after TT without a CIN. Organic contaminants are present on the surface of a zinc sample in the initial state. This primarily follows from the C1s electron spectrum. It can be described by peaks corresponding to saturated hydrocarbon chains (a maximum at 285.0 eV; full width at half maximum (FWHM) = 1.4 eV) and groups of atoms containing both carbon and oxygen (286.2, 288.6, and 289.4 eV, FWHM = 1.6 eV) [25].

The presence of organic contaminants is also confirmed by the peaks at 532.6 and 532.1 eV (FWHM 1.6 eV) in the oxygen O1s spectrum. The peak with a maximum at 533.0 eV (FWHM 1.6 eV) corresponds to adsorbed water. In the region of lower binding energies, the peaks of air-formed zinc hydroxide and oxide are observed in the spectrum. The maximum for Zn(OH)_2_ was at 531.7 eV (FWHM 1.7 eV) and that for ZnO was at 530.1 eV (FWHM 1.2 eV). The ZnL_3_M_45_M_45_ Auger spectrum of the sample in the initial state can be approximated by three peaks corresponding to the zinc metal lattice (494.1 eV), the air-formed oxide (495.8 eV), and zinc hydroxide (499.1 eV).

After TT, peaks corresponding to organic contaminants are recorded on the surface of the sample, similarly to the sample in the initial state. In addition to the states of carbon identified previously, yet another peak with a binding energy of 284.2 eV (FWHM 1.3 eV) appears in the spectrum. It can be interpreted as the presence of C=C moieties in the structure of organic contaminants. The XPS spectrum of oxygen and the XPS spectra of zinc show the same states as those for zinc before TT, but with a different ratio of peak areas.

**Figure 5:**
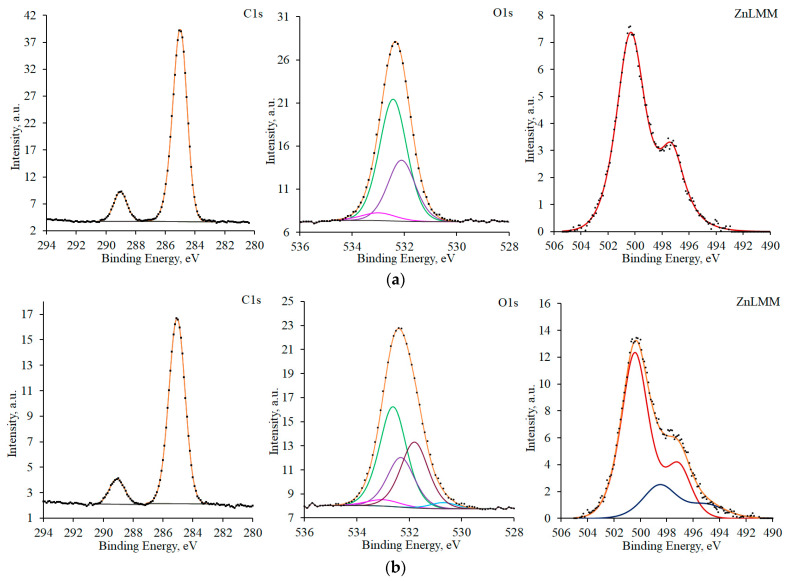
C1s and O1s XPS spectra, and ZnL_3_M_45_M_45_ Auger spectra for samples with *τ*_exp_ = 1 h (**a**) and 24 h (**b**). Spectral components are given in different colors for better visibility.

After the CT, no peaks corresponding to the spectra of the substrate were observed on the experimental curves (Figure 5). This is due to the fact that the thickness of the surface film formed in EHA vapors significantly exceeds the depth accessible to the method.

In the region of 280–294 eV, the samples with τ_exp_ = 1 h manifest two well-resolved peaks with maxima at 285.0 and 289.0 eV (FWHM 1.1 eV) that can be attributed to the carbon atoms of the hydrocarbon chain and the carboxyl group of EHA, respectively. The integral intensity ratio of the peaks was 7:1, which is consistent with the EHA stoichiometry (C_4_H_9_-CH(C_2_H_5_)-COOH).

The O1s oxygen spectrum is adequately described by three peaks. One of them, with a maximum at 533 eV and FWHM = 1.6 eV, can be attributed to adsorbed water vapor; the other two, with maxima at 532.1 and 532.4 eV (FWHM 1.3 eV), to the oxygen atoms of the hydroxyl and carboxyl groups, respectively. The integral intensity ratio of these peaks is 1:2. The ZnL_3_M_45_M_45_ Auger spectrum manifests two peaks. Such a composite spectrum shape is typical for Zn^2+^ compounds and corresponds to one state of zinc.

The spectra described above, with consideration for the sensitivity coefficients, allow us to conclude that the zinc surface after CT at τ_exp_ = 1 h contains a compound comprising seven carbon atoms of the hydrocarbon chain, one carbon atom and two oxygen atoms of the carboxyl group, and one hydroxyl oxygen atom per zinc atom. It is very likely that this compound is the basic salt of zinc and EHA, namely C_4_H_9_-CH(C_2_H_5_)-COO-Zn-OH.

The shape of the experimental curve, the positions of maxima, and the intensity ratio of carbon spectrum peaks did not change for samples with τ_exp_ = 24 h. This leads to the quite expected conclusion that the C_4_H_9_-CH(C_2_H_5_)- hydrocarbon radical did not undergo changes during air exposure of samples after CT.

In contrast to the spectra of carbon, those of oxygen and zinc changed for samples with τ_exp_ = 24 h. Upon exposure to air, the intensities increased in the regions of 529–532 eV for O1s and 491–499 eV for ZnL_3_M_45_M_45_. Two peaks with maxima at 530.7 eV (FWHM 1.24 eV) and 531.8 (FWHM 1.3 eV) appear on the oxygen spectrum of the sample with τ_exp_ = 24 h. The first one, whose maximum is shifted towards higher bond energies relative to ZnO, can be assigned to the Zn–O–Zn oxygen bridge. The second one corresponds to the oxygen atoms of carboxyl groups whose electron density differs from the groups described above.

In addition to the state determined at τ_exp_ = 1 h, the ZnL_3_M_45_M_45_ Auger spectrum for the sample with τ_exp_ = 24 h contains peaks with maxima at 498.6 and 495 eV (FWHM 3.14 and 3.54 eV, respectively). They can be assigned to the zinc atoms of the R-COO-Zn-O-Zn-OOC-R moiety.

Taking the sensitivity coefficients into account, these spectra allow us to conclude that the zinc surface after CT and with τ_exp_ = 24 h contains a compound with the following probable structure: C_4_H_9_-CH(C_2_H_5_)-COO-Zn-O-Zn-OOC-CH(C_2_H_5_)-C_4_H_9_. It can be assumed that this is a dehydration product of the basic salt of zinc and EHA.

Thus, the CT of zinc mainly produces the basic EHA salt (C_4_H_9_-CH(C_2_H_5_)-COO-Zn-OH). When the sample is exposed to air outside the chamber, this salt undergoes dehydration and is converted to a compound with the formula C_4_H_9_-CH(C_2_H_5_)-COO-Zn-O-Zn-OOC-CH(C_2_H_5_)-C_4_H_9_. Apparently, this transformation determines the increase in the protection efficiency.

## 4. Conclusions

1. EHA is a CIN of zinc corrosion that provides efficient protection in accelerated tests and under outdoor conditions.

2. As the temperature of zinc CT increases, the efficiency of EHA first increases and then decreases. The growth in the protective effect is due to an increase in the inhibitor’s vapor pressure that favors adsorption. The descending branch of the temperature dependence is due to the decrease in adsorption as the adsorbent (zinc) is heated. The optimum temperature of zinc CT with EHA vapors is around 100 °C.

3. The efficiency of EHA increases as the CT time increases to 1 h. This is the optimal duration for the system in question. Chamber treatment for a longer time does not give a positive effect and is inexpedient.

4. Upon chamber treatment of zinc under optimal conditions, adsorption films of EHA up to 100 nm thick are formed on the surface. A fraction of the acid reacts with the metal and the surface oxide during CT to form the basic salt C_4_H_9_-CH(C_2_H_5_)-COO-Zn-OH.

Once zinc has been removed from the chamber and exposed to open air, the basic salt is dehydrated and converted to a compound with the formula CH(C_2_H_5_)-COO-Zn-O-Zn-OOC-CH(C_2_H_5_)-C_4_H_9_ that is responsible for the metal protection. This process causes the growth in the protection efficiency during the first day of exposure of the metal outside the chamber at room temperature.

5. Protective adsorption films of EHA passivate the metal and stabilize its passive state. Their action is both due to shielding of the surface from the corrosive environment and to the inhibition of corrosion processes on the active metal surface.

## Figures and Tables

**Table 1:** Composition of Ts0 zinc alloy (mass%).

Zn	Pb	Cd	Fe	Cu	Sb	As	Al
>99.975	<0.013	<0.004	<0.005	<0.001	<0.001	<0.0005	<0.005

**Table 2:** PAE of adsorption films formed upon zinc treatment with vapors of individual CINs. Recurrent moisture condensation conditions. *t*_CT_ = 120 °C; *τ*_CT_ = 1 h; *τ*_exp_ = 24 h.

CIN	*τ*_cor_, h
Original state	1.0
TT without CIN	1.0
Octadecylamine	4.0
Diphenylguanidine	2.0
Polyethylene polyamine	12.0
Hexamethylenetetramine	5.0
Captax	4.0
Altax	2.0
Benzotriazole	8.0
Lauric acid	24.0
Tridecanoic acid	48.0
Stearic acid	24.0
Oleic acid	12.0
Linolenic acid	12.0
EHA	1680.0

**Table 3:** PAE of adsorption films formed during zinc treatment with EHA vapors at various *t*_CT_. Salt fog conditions. *τ*_CT_ = 1 h; *τ*_exp_ = 24 h.

Treatment Conditions	τ_pr_ at Various *t*_CT_, h				
	20 °C	60 °C	80 °C	100 °C	120 °C
TT without CIN	1.0	1.0	1.0	1.0	1.0
With EHA	1.0	48.0	60.0	72.0	65.0

**Table 4:** PAE of adsorption films formed upon zinc treatment with EHA vapors at various *τ*_CT_. Salt fog conditions. *t*_CT_ =100 °C; *τ*_exp_ = 24 h.

Treatment Conditions	τ_pr_ at Various *τ*_CT_ Values, h					
	5 min	15 min	30 min	60 min	120 min	180 min
TT without CIN	1.0	1.0	1.0	1.0	1.0	1.0
With EHA	20.0	28.0	56.0	72.0	72.0	72.0

**Table 5:** PAE of adsorption films formed upon zinc treatment with EHA vapors. Outdoor tests in an urban atmosphere. *t*_CT_ = 100 °C; *τ*_CT_ = 1 h; *τ*_exp_ = 24 h.

Treatment Conditions	τ_pr_, Months
Initial state	0.2
TT without CIN	0.2
With EHA	>42

**Table 6:** Effect of zinc treatment with EHA vapors on the characteristics of anodic polarization curves in BBS (pH 7.36, 1 mM NaCl). *t*_CT_ = 100 °C, *τ*_CT_ = 1 h.

No.	Treatment Conditions	*i*_pas_, μA/cm^2^	*E*_st_, V	*E*_br_, V
**1**	**Initial state**	**38**	**–0** **.7** **70**	**–0** **.505**
**2**	**TT without CIN,** ***τ*_exp_ = 24 h**	**15**	**–0** **.780**	**–0** **.4** **60**
3	EHA, *τ*_exp_ = 1 h	5	–0.750	–0.465
4	EHA, *τ*_exp_ = 5 h	–	–0.700	–0.405
**5**	**EHA, *τ*_exp_** **= 24 h**	–	**–0** **.735**	**–0** **.340**
6	EHA, *τ*_exp_ = 72 h	–	–0.650	–0.350

**Table 7:** Equivalent circuit parameters for various conditions of zinc treatment (BBS, pH 7.36, 1 mM NaCl), *t*_CT_ = 100 °C, *τ*_CT_ = 1 h.

No.	Treatment Conditions	*R*_s_, kΩ·cm^2^	*CPE*_sl_, S·s*^n^*/cm^2^	*n*_sl_	*R*_sl_, kΩ·cm^2^	*CPE*_dl_, S·s*^n^*/cm^2^	*n*_dl_	*R*_ct_, kΩ·cm^2^	Z, %
**1**	**Initial state**	**0.36**	**5.19·10^−6^**	**1**	**0.99**	**6.66 10^−5^**	**1**	**0.68**	**–**
**2**	**TT without CIN,** ***τ*_exp_ =24 h**	**0.14**	**2.37·10^−6^**	**0.92**	**1.13**	**1.94 10^−5^**	**0.81**	**0.77**	**12.11**
3	EHA *τ*_exp_ = 1 h	0.42	3.51·10^−6^	1	13.24	2.19·10^−5^	1	18.34	94.71
4	EHA *τ*_exp_ = 5 h	0.42	1.68·10^−6^	1	30.37	1.15·10^−5^	1	20.99	96.75
**5**	**EHA *τ*_exp_ = 24 h**	**0.49**	**1.10·10^−6^**	**1**	**49.21**	**6.88·10^−6^**	**1**	**45.49**	**98.24**
6	EHA *τ*_exp_ = 72 h	0.37	1.48·10^−6^	1	48.99	6.92·10^−6^	1	45.31	98.22

**Table 8:** Degrees of protection via the blocking (γ_sl_) and activation mechanisms (γ_ct_) for different modes of zinc CT, *t*_CT_ = 100 °C, *τ*_CT_ = 1 h. The values of γ were calculated with respect to zinc in the initial state.

No.	Treatment Conditions	*γ*_sl_	*γ*_ct_
**1**	**Initial** **state** **before** **TT**	**–**	**–**
**2**	**TT without CIN,** ***τ*_exp_ = 24 h**	**1.1**	**1.1**
3	EHA *τ*_exp_ = 1 h	13.4	27.0
4	EHA *τ*_exp_ = 5 h	30.7	30.8
**5**	**EHA *τ*_exp_ = 24 h**	**49.7**	**67.5**
6	EHA *τ*_exp_ = 72 h	49.5	56.6

**Table 9:** Effect of CT on the thickness of surface films formed in different modes of zinc CT, *t*_CT_ = 100 °C, *τ*_CT_ = 1 h.

No.	Treatment Conditions	Thickness, nm	
		Oxide Film	Adsorption Film
**1**	**Initial** **state** **before** **TT**	**–**	**–**
**2**	**TT without CIN,** ***τ*_exp_ =24 h**	**5.5**	**–**
3	EHA *τ*_exp_ = 1 h	1.0	105
4	EHA *τ*_exp_ = 5 h	1.0	95
**5**	**EHA *τ*_exp_ = 24 h**	**1.0**	**85**

## Data Availability

Not applicable.
